# Development of a quantitative COVID-19 multiplex assay and its use for serological surveillance in a low SARS-CoV-2 incidence community

**DOI:** 10.1371/journal.pone.0262868

**Published:** 2022-01-21

**Authors:** Cassandra Guarino, Elisabeth Larson, Susanna Babasyan, Alicia Rollins, Lok R. Joshi, Melissa Laverack, Lara Parrilla, Elizabeth Plocharczyk, Diego G. Diel, Bettina Wagner

**Affiliations:** 1 Department of Population Medicine and Diagnostic Sciences, College of Veterinary Medicine, Cornell University, Ithaca, NY, United States of America; 2 Cayuga Medical Center, Ithaca, NY, United States of America; Qatar University, QATAR

## Abstract

A serological COVID-19 Multiplex Assay was developed and validated using serum samples from convalescent patients and those collected prior to the 2020 pandemic. After initial testing of multiple potential antigens, the SARS-CoV-2 nucleocapsid protein (NP) and receptor-binding domain (RBD) of the spike protein were selected for the human COVID-19 Multiplex Assay. A comparison of synthesized and mammalian expressed RBD proteins revealed clear advantages of mammalian expression. Antibodies directed against NP strongly correlated with SARS-CoV-2 virus neutralization assay titers (r_sp_ = 0.726), while anti-RBD correlation was moderate (r_sp_ = 0.436). Pan-Ig, IgG, IgA, and IgM against NP and RBD antigens were evaluated on the validation sample sets. Detection of NP and RBD specific IgG and IgA had outstanding performance (AUC > 0.90) for distinguishing patients from controls, but the dynamic range of the IgG assay was substantially greater. The COVID-19 Multiplex Assay was utilized to identify seroprevalence to SARS-CoV-2 in people living in a low-incidence community in Ithaca, NY. Samples were taken from a cohort of healthy volunteers (n = 332) in early June 2020. Only two volunteers had a positive result on a COVID-19 PCR test performed prior to serum sampling. Serological testing revealed an exposure rate of at least 1.2% (NP) or as high as 5.7% (RBD), higher than the measured incidence rate of 0.16% in the county at that time. This highly sensitive and quantitative assay can be used for monitoring community exposure rates and duration of immune response following both infection and vaccination.

## Introduction

The community of Ithaca, NY (in Tompkins county) responded swiftly and decisively to the impending threat of SARS-CoV-2, closing public and private schools, most child daycare centers, as well as the local Colleges and University in March 2020. In addition, the community followed New York State guidance on the closure of non-essential businesses, and many individuals quickly adapted practices on social distancing, mask wearing and travel restrictions. As a result, the community spread of SARS-CoV-2 in Ithaca was greatly limited, while a severe outbreak occurred simultaneously in New York City, the initial epicenter of COVID-19 in the US, and its surrounding regions. From March 13 to June 30, the number of daily new cases in New York City reached 6364 (April 6, 2020), while in Tompkins county, daily case numbers peaked at 16 (March 27, 2020). During the first week of June, the duration of the seroprevalence study described here, New York City had a 7-day rolling average of 450 new cases/day, while the 7-day rolling average in Tompkins county was <1 new case/day [1, 2]. The availability of testing to identify active infections was crucial but limited in the first weeks and months of the pandemic. In addition, assays that could detect prior exposure to the SARS-CoV-2 virus, the causative agent of COVID-19 were initially not available.

Since our work on a serologic COVID-19 assay began, large research and development undertakings around the world have led to the development of a variety of different, but related, serologic assays. These serologic assays, many of which have received USA FDA Emergency Use Authorization, have been recently reviewed [3–5], and information on newly developed tests is available through multiple websites [6–8]. Each assay measures different components of the host immune response against SARS-CoV-2. For example, the different assays detect IgG [9–23], IgM [11, 13, 15–22], IgA [10, 13], or pan-Ig [24] specific for different recombinant SARS-CoV-2 antigens: full length spike protein (S) [15, 19, 22, 25], subunit 1 of S (aa14-685, S1) [10, 12, 13, 23], subunit 2 of S (aa686-1273, S2) [13, 23], the receptor binding domain (aa319-541, RBD) [12, 13, 21, 25], nucleocapsid protein (full length protein, NP) [9, 11–13, 15, 16, 19, 24, 25], and/or membrane protein (M) [13]. These assays utilize different techniques, including ELISA [10, 11, 16, 21, 25–28], rapid detection lateral flow qualitative assays [14, 17, 20, 22], singleplex chemiluminescent microparticle immunoassays [9, 15, 19, 23, 24, 29], or multiplex bead-based Luminex assays [12, 13, 30–38].

Community-wide seroprevalence analyses provide valuable insight into the breadth of community exposure to the virus in regions with rapidly increasing COVID-19 related hospitalizations. These studies have identified that approximately 0.1–6% [9, 14, 17, 18, 28, 29, 36, 39–45], and as high as 17–22.7% [20, 37, 46], of individuals in communities with rapidly increasing cases present SARS-CoV-2 specific IgG antibodies. However, many studies compared multiple assays on the same sample set [3, 12, 25, 47–61], and showed discrepancies in detection rate between tests. For example, one study compared six different assays on convalescent PCR-confirmed COVID-19 patients in Germany. The different assays detected specific antibodies in 66.7–89% of the samples, depending on the assay used [48]. Accurate detection of SARS-CoV-2 specific antibodies presents specific challenges in communities with low case incidence due to the decreased positive predictive value. For example, a comparison of three different assays on serum samples from a region of Japan with no identified SARS-CoV-2 outbreaks at the time testing was performed resulted in inconsistent seroprevalence values ranging from 0–3.3%, depending on which test was used [54]. This highlights the importance for high sensitivity and specificity in order to accurately detect rare seropositive cases amongst the broad population.

Here, we describe the development of a COVID-19 Multiplex Assay to simultaneously quantify antibodies directed against three SARS-CoV-2 antigens, NP, RBD, and S1, as indicators of previous SARS-CoV-2 exposure. We demonstrate the importance of the antigen expression methodology for assay performance, compare assay results to virus neutralization, and evaluate the detection of different antibody isotypes. Finally, we utilized this assay to perform surveillance of the non-diagnosed exposure rate in the Cornell University community residing in Ithaca, NY in early June 2020.

## Materials and methods

### Samples for assay validation

De-identified serum samples (n = 78) from a previous serologic study conducted in 2019, collected in Binghamton, NY prior to SARS-CoV-2 being introduced in the United States, were used as control serum samples (pre-COVID-19) to investigate non-specific reactivity of assay components–all samples were from participants that indicated willingness to have their samples used for future research. All samples for this control group originated from people living in upstate NY in 2019 and included participants 20–75 years of age (median: 55.5 years), including 18.3% male and 61.7% female volunteers.

De-identified convalescent human serum samples from PCR confirmed SARS-CoV-2 infected individuals (n = 20) were obtained from Cayuga Medical Center (CMC, IRB protocol 0420EP), including samples from 10 non-hospitalized patients in the Cayuga Health System, with three serial samples from two patients, and two serial samples from six patients. Individuals were 16–72 years of age (median: 40 years) and were 70% male and 30% female. ROC curve analysis, described below, used only the first sample from individuals contributing multiple samples. Details of clinical history, sampling dates, and diagnostic criteria were not provided with these samples. One additional de-identified convalescent sample from a PCR confirmed SARS-CoV-2 infected individual was provided by Cornell Weill Medicine (WMC). Additional unique de-identified convalescence serum samples (n = 30), >21 days post SARS-CoV-2 PCR positive nasopharyngeal swab result, were obtained from the NYS Department of Health (NYSDOH), Wadsworth Center. These samples were from individuals residing in NYS, and additional demographic information was not provided.

### Virus neutralization assay

A virus neutralization assay [62, 63] was performed to assess the levels of neutralizing antibodies in serum. All serum samples were heat inactivated at 56°C for 30 minutes prior to the virus neutralization assay. Each sample was serially diluted (2-fold dilutions, 1:8 to 1:1024) and incubated with 100 TCID_50_ of SARS-CoV-2 Hu-WA-1 strain (GenBank: MN985325.1) for 1h at 37°C. Following incubation, 100 μl of a cell suspension of Vero CCL-81 cells was added to each well of the 96-well plate and incubated for 72h at 37°C with 5% CO_2_. Virus neutralization assays were read under an inverted microscope using SARS-COV-2 cytopathic effects as an indicator. Neutralizing antibody titers were expressed as the reciprocal of the highest dilution of serum capable of completely inhibiting SARS-CoV-2 infection/replication. Negative and positive human control sera were included in all assays. All samples were tested in duplicate and the endpoint titer determined based on the highest dilution of serum that presented 100% neutralization against SARS-CoV-2. The reciprocal titer of the highest serum dilution with 100% neutralization is presented.

### SARS-CoV-2 antigens

A synthetic peptide of the RBD region of the Spike protein (synthetic RBD) was a commercially available (LifeTein LLC, Somerset, NJ, product number LT5587). This 74 amino acids peptide (aa 433 to 506, YP_009724390) was composed of the following sequence: NH2- VIAWNSNNLDSKVGGNYNYL YRLFRKSNLKPFERDISTEI YQAGSTPCNGVEGFNCYFPL QSYGFQPTNGVGYQ- CONH2, including a disulfide bridge: Cys480-Cys488.

NP, RBD and S1 SARS-CoV-2 antigens expressed from mammalian cell culture were produced using a previously described IL-4 fusion protein expression system [64]. The SARS-CoV-2 antigens were cloned from SARS-CoV-2 RNA derived from Vero cell cultures infected with SARS-CoV-2 strain Hu-WA-1. cDNA was synthesized using the SuperScript III Reverse Transcriptase (Life Technologies) and oligo dT and six hexamer random primers following the manufacturer’s recommendations. The cDNA was used as template to amplify and clone nucleotide sequences, corresponding to the partial N-terminal domain of the S1 subunit without the signal peptide (aa 16 to 264, YP_009724390), RBD (aa 319 to 529, YP_009724390) of the Spike protein, and the whole NP antigen (aa 1–419, YP_009724397). Platinum SuperFi DNA Polymerase was used for amplification as per manufacturer’s instructions (ThermoFisher Scientific, Waltham, MA, USA). The primer sequences are summarized in Table 1. The PCR products were cloned into the multiple cloning site of mammalian expression vector pcDNA3.1 (ThermoFisher Scientific, Waltham, MA, USA) 3’ of the horse IL-4 sequence, as described previously [64].

**Table 1:** Primer sequences for expression cloning SARS-CoV-2 antigens.

Antigen	Forward primer ^a^	Reverse primer ^b^
**S1**	AAGGATCCTGTTAATCTTACAACCAGAACTCAATT	AAAGGTACCTTAGCTGCAGCACCAGCTGTCCAACCTG
**RBD**	AAGGATCCAAGAGTCCAACCAACAGAATCTATTGTT	AAAGGTACCTTACTTTTTAGGTCCACAAACAGTTGCT
**NP**	AAGGATCCAATGTCTGATAATGGACCCCAAAATC	AAAGGTACCTTAGGCCTGAGTTGAGTCAGCACTG

^a^ *BamHI* restriction site for cloning is underlined

^b^ *KpnI* restriction site for cloning is underlined.

The sequences of RBD and NP were identical to Spike protein and nucleocapsid protein sequences, respectively, of Wuhan-Hu-1 isolate (NC_045512), as verified by Sanger sequencing. S1 had a deletion of 10 amino acids, position 67–77 and was identical to the sequence of GenBank entry, accession number MT772569.

CHO-K1 cells were transiently transfected with recombinant expression constructs. Geneporter II transfection reagent was used per manufacturer’s instructions (Genlantis, San Diego, CA, USA). F12 medium (Gibco, Gaithersburg, MD), supplemented with 10% Fetal bovine serum (Atlanta Biologicals, Flowery Branch, GA) was used as growth medium. Expression and secretion of the recombinant SARS-CoV-2 antigen fusion proteins by CHO transfectants was confirmed using the IL-4 tag by flow cytometric analysis and ELISA, respectively, as previously described [64]. After 24–30 hours of incubation, the supernatants were harvested for fluorescent bead-based multiplex assays.

### Fluorescent bead-based assays

Fluorescent beads (MicroPlex®, Luminex Corp., Austin, TX, USA) were coupled as previously described [65]. Briefly, the SARS-CoV-2 proteins expressed as IL-4 fusion proteins were bound to the beads through an anti-IL-4 antibody, clone 25 (RRID: AB_2737308) by incubating the anti-IL-4 coupled beads with the fusion protein supernatant solution for 30 minutes at room temperature, followed by a wash step. The synthetic RBD peptide was directly coupled to beads. The multiplex assay was performed as previously described [66], with a few modifications. Briefly, beads were incubated with serum samples diluted 1:10, 1:50, or 1:100, and bound serum antibodies were detected with biotinylated anti-human IgG (H+L), ‘pan-Ig’ (RRID: AB_2337628), diluted 1:10,000. Alternatively, the assay was detected with one of the following biotinylated antibodies for isotype detection: anti-human IgG, Fcɣ fragment specific, ‘IgG’ (RRID: AB_2337630), anti-human IgM, Fc5μ specific, ‘IgM’ (RRID: AB_2337632), or anti-human serum IgA, α-chain specific, ‘IgA’ (RRID: AB_2337624). All isotype detection reagents were diluted 1:10,000. A serum dilution of 1:100 was found to be optimal. The Luminex platform allows for quantification over a large range, from approximately 100–25,000 MFI. While the MFI values do not provide an absolute antibody concentration, the MFI values reflect the quantity of antigens-specific antibody in the sample, with a dynamic range that exceeds that of a typical ELISA.

### Samples for serologic surveillance

Samples for serologic surveillance were collected at Cornell University at a ‘COVID-19 Healthy Volunteer Clinic’ (COVID-19 HVC) performed during the first week of June 2020. Eligible volunteers were non-pregnant healthy adults, 18–78 years of age, >110lbs, and members of the Cornell University community in Ithaca, NY, including faculty, staff, and students, or their direct family members. Volunteers answered a brief survey, including travel and health history since January 2020, and donated samples of blood and saliva. This project was approved by the Institutional Review Board for Human Participants at Cornell University, Protocol ID# 2004009584.

### Statistical analysis

Confidence intervals for sensitivity and specificity were calculated as “exact” Clopper-Pearson confidence intervals. Spearmen nonparametric correlation coefficients, r_sp_, were calculated with one-tailed *p* values. One-way ANOVAs were performed with Dunnett’s multiple comparison tests. Data were considered significant if *p* values were < 0.05. Statistical analyses, including ROC curve analysis, were performed using Prism GraphPad version 6.0.

## Results

### Antigen selection

**Fig 1:**
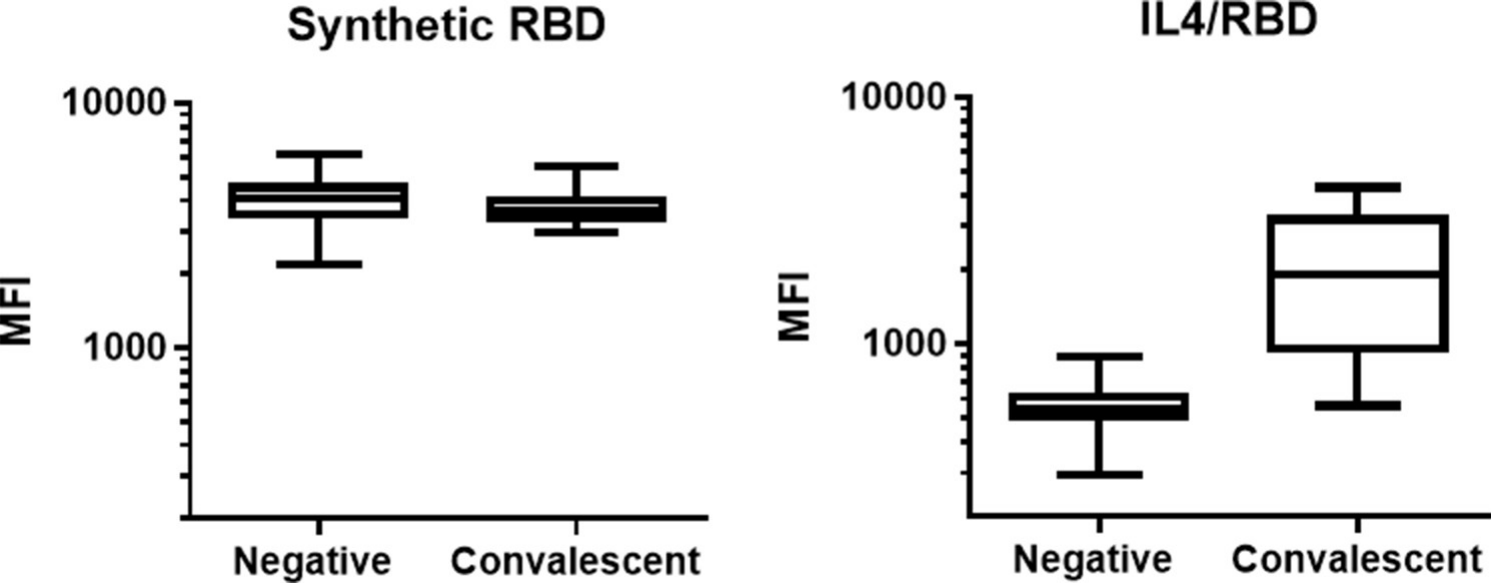
Comparison between fluorescent bead COVID-19 assays using synthetic RBD or mammalian expressed IL-4/RBD fusion protein as antigens for serologic testing. Negative serum samples (n = 78) were taken in 2019 prior to the COVID-19 pandemic. Convalescent serum samples (n = 21) were obtained from COVID-19 patients (n = 11). The plots represent pan-Ig median fluorescence intensity (MFI) values.

With the goal to identify the best antigens for the COVID-19 Multiplex assay, different proteins of SARS-CoV-2 were evaluated and compared as antigen targets for a serological multiplex assay using a pan-Ig detection antibody. Initially, a commercially available synthetic RBD peptide of the Spike protein and the full-length RBD expressed in mammalian cells as an IL-4 fusion protein were compared using a sample set of pre-COVID-19 negative serum samples as ‘controls’ (n = 78) and convalescent samples from CMC and WCM as ‘patients’ (n = 21). The assay based on synthetic RBD resulted in an area under the ROC curve (AUC) of 0.6496 (95% CI: 0.5256–0.7735), indicating poor discrimination between patients and controls. The AUC for IL-4/RBD evaluated on the same sample set was 0.9945 (95% CI: 0.9830–1.006), indicating excellent discrimination between patients and controls. The IL-4/RBD assay also generated a wider dynamic range of pan-Ig MFI results in comparison to the assay with synthetic RBD antigen (Fig 1). These results supported the use of the mammalian expressed IL-4/RBD antigen for serological assay development.

Additional SARS-CoV-2 antigens expressed in the mammalian cells as IL-4 fusion proteins included S1, NP, M and E proteins. Each antigen was first screened in individual assays with a subset of convalescent serum samples and negative samples for pan-Ig (S1 Fig). The IL-4/NP and IL-4/S1 antigens together with IL-4/RBD were selected for further evaluation.

### Correlation to virus neutralization

**Fig 2:**
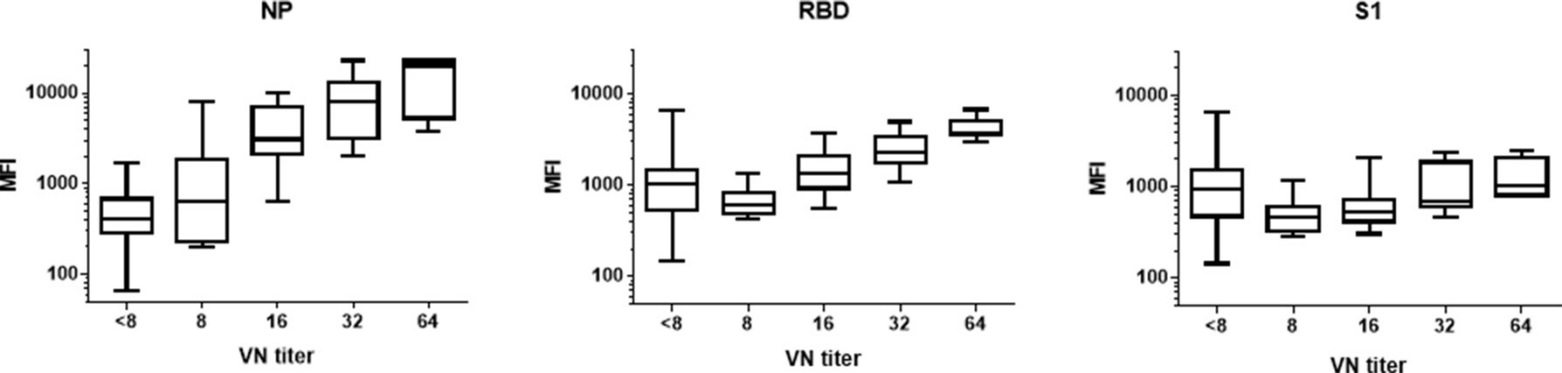
Correlation of virus neutralization titers with COVID-19 multiplex results using NP, RBD and S1 antigens. The SARS-CoV-2 antigens were expressed in mammalian cells as IL-4 fusion proteins, and the three antigens were multiplexed. Serum samples included samples taken pre-COVID-19 (n = 7), obtained from healthy people in the first week of June 2020 (COVID-19 HVC, n = 50), and from convalescent patients (n = 51). The correlation coefficients, r_sp_, for NP, RBD and S1 were 0.726, 0.436, and -0.055, respectively.

Serum samples from pre-COVID-19 controls (n = 7), from healthy people collected in the first week of June 2020 (COVID-19 HVC, n = 50), and from convalescent patients (n = 50) were evaluated for antibodies (pan-Ig) directed against NP, RBD and S1 in the COVID-19 Multiplex Assay. Each of these serum samples was also tested for neutralizing antibodies. Pan-Ig directed against NP highly correlated with neutralizing antibodies (r_sp_ = 0.726; *p* < 0.0001). RBD antibodies correlated moderately (r_sp_ = 0.436, *p* < 0.0001), while those directed against S1, where the version of S1 expressed contained a 10 amino acid deletion, did not correlate with neutralizing antibodies (r_sp_ = 0.055, *p* = 0.2852) (Fig 2).

Overall, serum neutralizing antibodies in convalescent patient samples were low and not reaching titers above 64. All convalescent samples from CMC (n = 20) had neutralizing antibody titers between 12 and 64. Convalescent serum samples taken >21 days after a positive PCR result (n = 30) generated variable virus neutralization results: thirteen had titers ranging from 16 to 64, six had a titer of 8, and the remaining eleven did not neutralize virus (<8). Of the 50 serum samples from the COVID-19 HVC, 41 serum samples did not neutralize virus, six had a neutralizing antibody titer of 8, and one, from an individual reporting a positive SARS-CoV-2 PCR result, had a titer of 32.

### SARS-CoV-2 specific antibody isotypes

**Fig 3:**
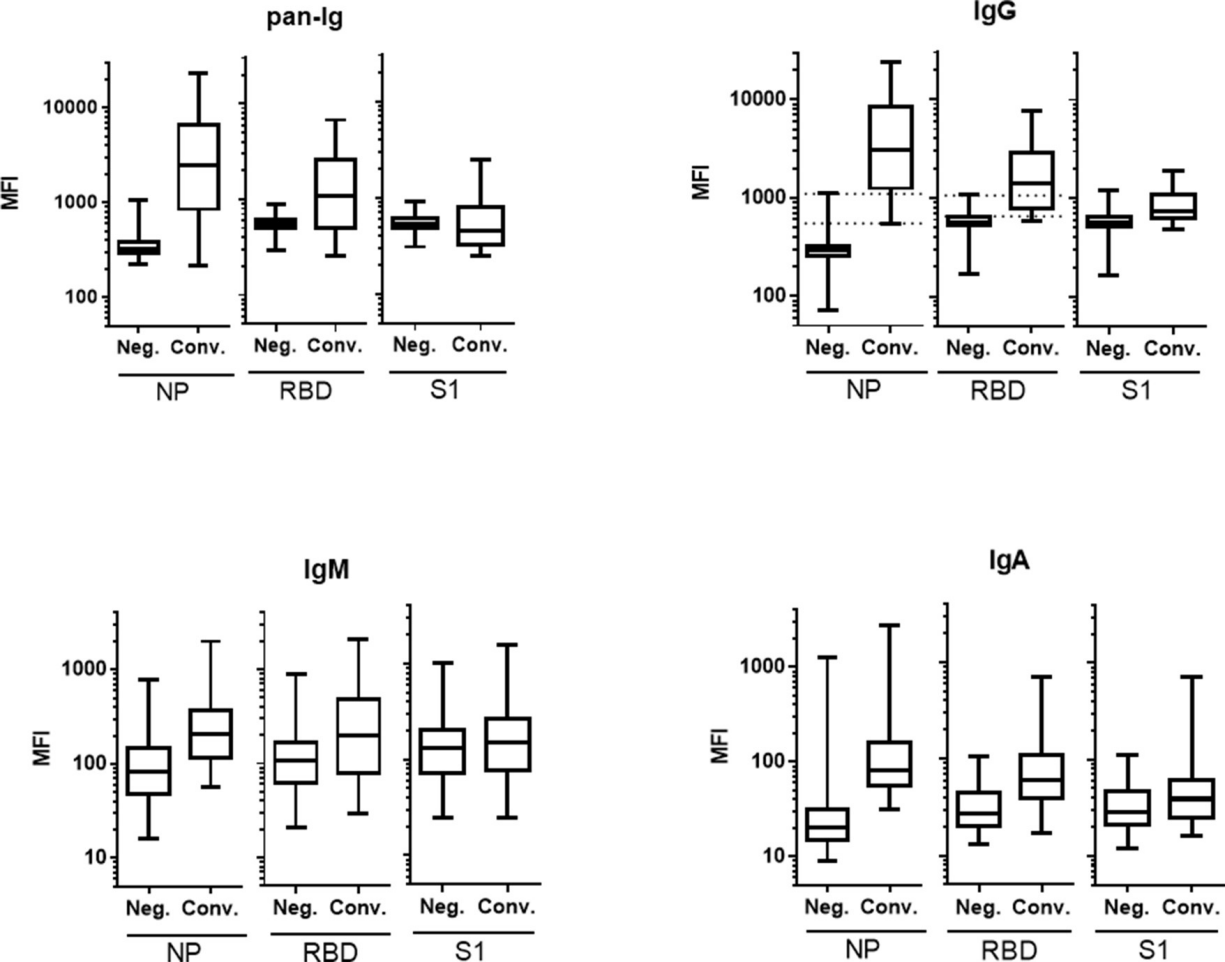
Comparison of antibody isotypes. The SARS-CoV-2 antigens, NP, RBD, and S1, were multiplexed in each assay run. Serum samples included samples taken pre-COVID-19, ‘Neg.’, (n = 78), and samples from convalescent patients, ‘Conv.’ (n = 41). The assays were detected with antibodies against either pan-Ig, IgG, IgM or IgA. Dashed lines represent the positive (upper line) and negative (lower line) cut-off values for RBD and NP IgG MFI. Values between the lines are considered ‘equivocal’.

Convalescent serum samples (n = 41) and pre-COVID-19 control serum (n = 78) used above for the pan-Ig measurement were also tested for IgG, IgM, and IgA isotypes in COVID-19 Multiplex assays. ROC curve analyses were performed on all data. The NP antigen resulted in outstanding discrimination between the convalescent patient and control groups, with AUCs above 0.90, on pan-Ig and all isotypes tested (Table 2). RBD yielded outstanding performance (AUC >0.90) with IgG and IgA, while the S1 assay showed only moderate performance with any isotype tested, leading to the exclusion of the S1 assay in the final COVID-19 Multiplex Assay. At a 1:100 serum dilution, the dynamic MFI range was greatest with NP for all isotypes tested. Detection with pan-Ig and IgG reagents produced a substantially greater dynamic range of results (50 to 24000 MFI) than with anti-IgM and IgA (Fig 3 and S2 Fig). The upper range of IgM and IgA values did not exceed 3,000 MFI on any antigen at a 1:100 serum dilution, limiting the value of these detection antibodies for low positive samples. Based on the comparison of three SARS-COV-2 antigens and four detection antibodies, the NP and RBD antigens in combination with the IgG detection reagent were selected for the final COVID-19 Multiplex assay.

**Table 2:** Area under the ROC curve (AUC) for each SARS-CoV-2 antigens and detection antibodies in the COVID-19 multiplex assay ^a^.

		Antigen ^b^		
		IL-4/NP	IL-4/RBD	IL-4/S1
**Detection antibody** ^**c**^	**pan-Ig**	**0.9432** (0.8893–0.9972)	0.7014 (0.5751–0.8277)	0.5876 (0.4517–0.7234)
	**IgG**	**0.9950** (0.9881–1.002)	**0.9368** (0.8971–0.9765)	0.7999 (0.7190–0.8807)
	**IgM**	**0.9005** (0.8394–0.9616)	0.7756 (0.6413–0.9100)	0.5162 (0.3518–0.6806)
	**IgA**	**0.9460** (0.9019–0.9901)	**0.9023** (0.8340–0.9712)	0.7027 (0.5678–0.8375)

^a^ ROC curve analysis was performed with serum from 41 convalescent patients and control serum samples from 78 pre-COVID-19 study participants. AUC is presented with 95% CI in parentheses.

^b^ Three antigens were multiplexed for each detection antibody assay run.

^c^ Each assay was detected with one of the four anti-human detection antibodies.

### Assay sensitivity and specificity of the COVID-19 multiplex assay

The sensitivity and specificity were determined for the optimized COVID-19 IgG assay based on the ROC analysis of convalescent serum samples from 41 individual patients and control serum samples from 78 pre-COVID-19 study participants. The assay’s lower and upper cut-off values, respectively, resulted in diagnostic sensitivity of >98%, and specificity of >95% for both antigens (Table 3). Sample results between the small lower and upper cut-off value ranges are considered equivocal and describe either high negative or low positive serum samples. Overall, IgG antibody measurement provides a wide dynamic range above the upper cut-offs for both antigens, allowing for optimal quantification of SARS-CoV-2 antibodies in positive samples (Fig 3).

**Table 3:** Sensitivity and specificity for the IgG COVID-19 multiplex assay ^a^.

		Cut-off values	Sensitivity^c^		Specificity^d^	
		Lower/Upper	%	95% CI	%	95% CI
**Antigen** ^**b**^	**NP**	555/1100	97.6	87.14% to 99.94%	100	95.38% to 100.0%
	**RBD**	650/1060	95.2	76.18% to 99.88%	98.7	93.06% to 99.97%

^a^ Sensitivity and specificity were determined from serum samples from 41 convalescent patients and 78 pre-COVID-19 study participants.

^b^ Antigens were expressed as IL-4 fusion proteins and were run in a two-plex assay.

^c^ Sensitivity was determined based on the lower cut-off values.

^d^ Specificity was determined based on the upper cut-off values.

### Community exposure rate

By June 1^st^ 2020, a total of 160 SARS-CoV-2 infection cases were reported in Tompkins County since the beginning of the pandemic, comprising approximately 0.16% of the county population [1]. Serum samples from 332 volunteers were taken during a clinic (COVID-19 HVC) between June 1–5, 2020 and were tested for IgG antibodies against NP and RBD in the COVID-19 Multiplex Assay. Serum samples from four individuals (1.2%) were positive on both NP and RBD, indicating evidence of previous infection with SARS-CoV-2 (Table 4). Of those four NP and RBD positive samples, only one individual was previously diagnosed by a COVID-19 positive PCR test. A second participant reported a positive PCR result prior to the COVID-19 HVC but did not have antibodies by the time the serum sample was taken. An additional 15 samples were positive on the RBD antigen only, but equivocal (n = 10) or negative (n = 5) on the NP antigen assay. By considering a positive result on one or both antigens as an indicator of previous infection with SARS-CoV-2, a total of 19 participants (5.7%) were identified as ‘seropositive’.

**Table 4:** Serum samples from healthy volunteers (n = 332) were tested for NP and RBD-specific IgG antibodies using the COVID-19 multiplex assay.

		NP		
		Positive	Equivocal	Negative
**RBD**	Positive	4	10	5
	Equivocal	0	0	6
	Negative	0	2	305

Self-reported illness history from the preceding 6 months was provided by each COVID-19 HVC participant and included dry cough (n = 77), fever (n = 54), GI upset (diarrhea) (n = 52), and loss of sense of smell or taste (n = 15). There was no significant difference between serum IgG values for either antigen for each reported illness history group, as compared to those who did not report any illness (Table 5).

**Table 5:** Comparison of NP and RBD-specific serum IgG antibodies in healthy volunteers by illness and travel history between January and May 2020.

Survey Response	n	antigen	Antibodies Median MFI (range)	p^a^
No illness	215	NP	387 (256–8069)	NA
		RBD	593 (415–8921)	NA
Dry cough	77	NP	417 (94–1505)	n.s.
		RBD	625 (124–4107)	n.s.
Fever	54	NP	403 (94–1158)	n.s.
		RBD	616.5 (124–1719)	n.s.
GI upset	52	NP	403 (248–1646)	n.s.
		RBD	608 (428–3054)	n.s.
Loss of smell/taste	15	NP	434 (94–1158)	n.s.
		RBD	694 (124–1365)	n.s.
No travel	206	NP	389 (254–8069)	NA
		RBD	600.5 (415–8921)	NA
Travel to NYC	49	NP	386 (248–1646)	n.s.
		RBD	595 (453–4796)	n.s.
Domestic Travel	103	NP	395 (94–2461)	n.s.
		RBD	632 (124–7047)	n.s.
International Travel	26	NP	397.5 (94–2461)	n.s.
		RBD	700 (124–7047)	< 0.05

^a^ P-values for illness groups compared to ’No illness’, and for travel groups compared to ’No travel’. Alpha = 0.05; n.s. = not significant; NA = not applicable.

Self-reported travel history from the preceding 6 months was also provided by each COVID-19 HVC participants and included travel to NYC (n = 49), domestic travel outside of NYS (n = 103), and international travel (n = 26). NP-specific IgG values in serum between groups with different travel history were similar to those without travel history. RBD-specific IgG values were significantly increased in the group with international travel history as compared to those who did not report any travel history (*p* < 0.05) (Table 5).

## Discussion

Development of a SARS-CoV-2 specific serological assay that can accurately quantify virus-specific antibodies in high and low COVID-19 incidence communities is crucial for assessing exposure rates to the virus, immune responses to vaccination, and the longevity of antibodies after infection or vaccination. Here, we described the validation of a new COVID-19 Multiplex Assay based on the SARS-CoV-2 RBD and NP antigens with excellent accuracy of detection, sensitivity and specificity, and a broad quantification range. In addition, the assay was used to detect undiagnosed prior infection with SARS-CoV-2 in individuals with no or mild disease in a low incidence community in Ithaca, NY.

Antibody responses against viral pathogens are polyclonal and typically directed against several immunogenic structures including conformational and linear epitopes on these antigens. The choice, quality and conformation of an antigen highly influences specificity, sensitivity, and the overall performance of the serological assay. Here, we compared several SARS-CoV-2 antigens to identify the optimal antigens for a sensitive fluorescent bead-based multiplex assay, including two different RBD antigens. One was a synthetic, commercially available peptide. The second RBD antigen was expressed in mammalian cells, resulting in the closest homolog to expression of the natural viral protein after infection of a human host. The serological assay based on mammalian RBD antigen resulted in excellent sensitivity and specificity and a wide linear quantification range for antibodies in serum, while the assay based on the synthetic RBD peptide had a narrow quantification range and a poor ability to distinguish pre-COVID-19 sera without antibodies against SARS-CoV-2 from COVID-19 patient sera. The serological results suggest that the synthetic RBD peptide had a low structural homology to the natural viral RBD protein. In addition, the commercial peptide was truncated (74 aa) compared to the 210 aa mammalian expressed RBD antigen, possibly leading to missing antigenic epitopes of the synthetic peptide.

For the final COVID-19 Multiplex Assay both antigens, NP and RBD, were thus produced in mammalian cells. Antibodies against NP highly correlated with neutralizing antibodies against the SARS-CoV-2 strain Hu-WA-1. Testing of pre-COVID-19 sera that were collected before the pandemic reached the US, confirmed that these samples had no reactivity with the NP antigen assay. Antibodies against SARS-CoV-2 NP in convalescent patient serum illustrated the wide dynamic quantification range of the NP assay. Overall, the NP assay had an outstanding diagnostic sensitivity and specificity of 97.6% and 100%, respectively. For comparison, the EUROIMMUN SARS-CoV-2 anti-spike S1 ELISA, a widely distributed pre-existing serologic assay for SARS-CoV-2, resulted in sensitivity values ranging from 67.0–97.0% and specificity values ranging from 96.0–100% [6, 10, 27, 28, 48–50, 55]. The USA Food and Drug Administration has independently evaluated many of the current COVID-19 serologic assays together on the same sample set, allowing a useful comparison of test accuracies [67]. Most of the current serologic assays for SARS-CoV-2 antibodies are validated with blood samples collected in the first 2–7 weeks after symptom onset [9, 10, 19, 21–25, 27–29, 39, 47, 50, 52, 53, 55, 59].

Two sets of convalescent sera were used for validation of the COVID-19 Multiplex Assay described here and included samples collected in Ithaca, NY within the first 4–6 weeks after clinical cases were first confirmed in the region (n = 11), and samples obtained from the NYS DOH from patients >21 days after a positive SARS-CoV-2 PCR result from a nasopharyngeal swab (n = 30). Other patient information, including duration and onset of symptoms and severity of disease was not available for these sample sets. It has been suggested that the magnitude of SARS-CoV-2-specific antibodies in serum is positively correlated with disease severity [68, 69] and antibodies wane rapidly, possibly within as little as three months after virus clearance [70–72]. Therefore, sensitivity may have been overestimated in assays that were validated using only early convalescent sera from hospitalized patients. Within our sample set, patients who had mild illness, or patients who were infected many months prior to testing may have had low serum antibody at the time the sample was taken, but SARS-CoV-2 specific antibodies could still be detected by the COVID-19 Multiplex Assay above the lower cut-off values.

Most serological COVID-19 assays described in the literature identified IgG or IgM antibodies against SARS-CoV-2 in patient serum, and a few have also investigated IgA antibodies [73, 74]. Here, we compared serum pan-Ig, IgG, IgM and IgA antibodies on the same assay platform indicating that measurement of IgG antibodies against SARS-CoV-2 most effectively differentiated convalescent patient serum from pre-COVID-19 serum. Measurement of NP- and RBD-specific serum IgA antibodies also distinguished known positive and negative sera, however serum IgA measurements were limited in magnitude and dynamic range. The ability of the COVID-19 Multiplex Assay to measure both IgG and IgA isotypes will enable further study of the immune response to SARS-CoV-2, including the quantification of antibody responses after vaccination. Furthermore, the COVID-19 Multiplex Assays for IgG and IgA will be valuable tools for measuring mucosal antibody responses due to the improved analytical sensitivity of the bead-based platform compared to conventional ELISA [75] and as shown previously for other respiratory viral pathogens [65, 76, 77].

We next used the COVID-19 Multiplex Assay to evaluate seroprevalence in healthy people in Ithaca, NY in June 2020. The confirmed COVID-19 incidence rate in the community at that time was low (0.16%). Out of 332 COVID-19 HVC participants a total of 19 (5.7%) had IgG antibodies against SARS-CoV-2 NP and/or RBD antigens. Based on the self-reported clinical COVID-19-like symptoms that the majority of the participants experienced since January 2020, it can be assumed that at least some of these individuals were infected with SARS-CoV-2 sometime earlier in the year. Many of the participants also travelled in the first 2.5 months in 2020 either internationally or to NYC which was the initial hot-spot of the COVID-19 pandemic in the US. Nevertheless, the severity of their symptoms did not advance to COVID-19 PCR testing or hospitalization and it can be assumed that several participants of the COVID-19 HVC did mount low and short-lasting antibody responses against the virus which were overall low again in June 2020. At the time of antibody testing, there was only an increased prevalence of RBD antibodies in serum from individuals with international travel history during the early months of the pandemic. Overall, the 5.7% seroprevalence rate was substantially higher than the confirmed infection rate of 0.16% suggesting that undiagnosed infections were prevalent in this community in the early months of the pandemic.

This highly sensitive and specific assay is a valuable tool for monitoring immune response to SARS-CoV-2 infection in both individuals and at the population level, however there are several limitations to the use of this serological assay. One potential limitation of this assay is the possibility for cross-reactivity with other known coronaviruses, in particular NP, which has moderate sequence homology with the nucleocapsid proteins of other human coronaviruses [78]. However, non-specific reactions against this antigen were not detected in our control group. Another potential limitation is that, in populations with low disease prevalence, as in the case of the population surveyed here, the positive predictive value of individual results will be low.

In conclusion, the use of the quantitative serological COVID-19 Multiplex Assay, for monitoring community exposure rates and obtaining more information on antibody magnitude and longevity after SARS-CoV-2 infection and vaccination, will help to assess infection risks in populations and individuals.

## Supporting information

S1 Fig Representative pan-Ig antibody values directed against NP, RBD, envelope protein (E) or membrane protein (M) of SARS-CoV-2 in the pools produced for internal assay standards. These pools included Negative (N), Low Positive (L), and High Positive (H) pooled samples.
(TIF)

S2 Fig IgA antibody values against NP and RBD for negative (“-“, n = 8) and positive (“+”, n = 15) serum samples measured at three different serum dilutions, 1:10, 1:50, or 1:100. (TIF)
