# Supplementary figures and images for: Development of a quantitative COVID-19 multiplex assay and its use for serological surveillance in a low SARS-CoV-2 incidence community
Source: PLoS One. 2022 Jan 21;17(1):e0262868. doi: 10.1371/journal.pone.0262868 (PMC8782306; doi:10.1371/journal.pone.0262868)

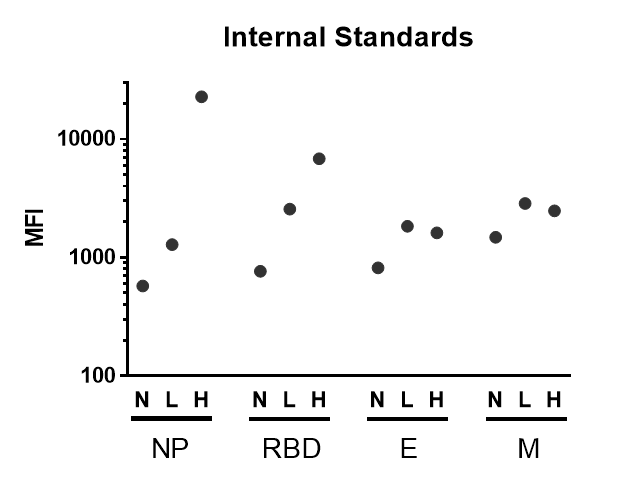

Supplement: S1 Fig — These pools included Negative (N), Low Positive (L), and High Positive (H) pooled samples. (TIF) [file pone.0262868.s001.tif]

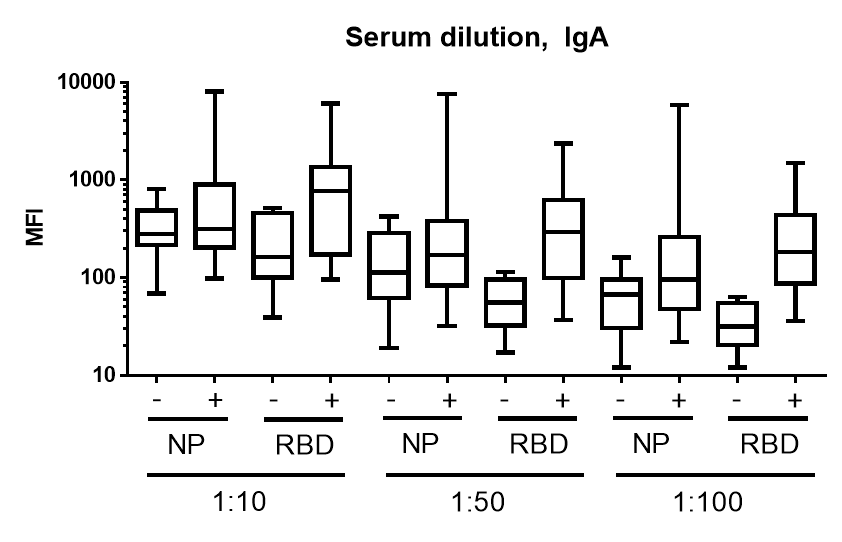

Supplement: S2 Fig — (TIF) [file pone.0262868.s002.tif]
